# Vertical transmission of Orf virus in goats and its prevention

**DOI:** 10.1186/s13567-026-01714-0

**Published:** 2026-02-25

**Authors:** Ming Pang, Furqan Munir, Jin Yao, Tianxing Wang, Shaofei Li, Yiming Chen, Ying Wen, Dekun Chen, Jun Liu, Wentao Ma

**Affiliations:** 1 Key Laboratory of Animal Biotechnology of the Ministry of Agriculture of China, College of Veterinary Medicine, Northwest Agriculture and Forestry University, Yangling, 712100 Shaanxi China; 2 State Key Laboratory of Plateau Ecology and Agriculture, Qinghai University, Xining, 810016 Qinghai China; 3 College of Agriculture and Animal Husbandry, Qinghai University, Xining , 810016 Qinghai China

**Keywords:** Orf virus, goats, vertical transmission, inactivated vaccine

## Abstract

Contagious ecthyma dermatitis, caused by Orf virus (ORFV), is an important zoonotic disease of small ruminants, particularly goats and sheep, and can occasionally infect humans. The morbidity of the disease is high in newborn kids or lambs as compared with adult animals, even leading to death in severe cases due to emaciation or secondary infections. Despite its significance, the precise route of ORFV transmission to newborn kids or lambs has remained unclear. In this study, we hypothesized that ORFV may be transmitted transplacentally from dam to fetus and investigated the presence of the virus in maternal–fetal interface tissues. Molecular detection targeting the ORFV *B2L* gene provided evidence supporting vertical transmission. The average viral loads in placental cotyledons and umbilical cords were 10^4.25^ copies/mg and 10^3.50^ copies/mg, respectively. Furthermore, vaccination of pregnant goats significantly reduced the vertical transmission rate of ORFV, with the ORFV-positive rate decreasing from 90.0% in non-vaccinated dams to 56.14% in vaccinated dams, from 71.43% to 14.29% in umbilical cord blood, and from 36.67% to 0% in kids born to vaccinated dams. Collectively, these findings identify vertical transmission as an important, previously under-recognized route of ORFV infection and highlight the importance of maternal vaccination in mitigating infection risk among newborn kids or lambs.

**Supplementary Information:**

The online version contains supplementary material available at 10.1186/s13567-026-01714-0.

## Introduction

Sheep and goat farming play a pivotal role in China’s agricultural economy and rural livelihoods, providing milk, meat, leather, fleece, and wool for human consumption. China accounted for 31.1% of global mutton and goat meat production in 2022 [1], with small ruminant farming playing an important role in arid, semi-arid, and mountainous regions where harsh climates and limited arable land make crop cultivation difficult [2]. The Chinese government and the Food and Agriculture Organization have supported breeding initiatives, further highlighting the importance of small ruminant production for agricultural modernization [3]. Therefore, maintaining animal health is essential for sustaining productivity and the long-term development of the small ruminant industry.

Contagious ecthyma dermatitis is an exanthematous pustular disease caused by Orf virus (ORFV). The disease is characterized by proliferative lesions that primarily affect the lips, oral mucosa, mammary glands, and genital regions. The incidence of Orf disease has been reported in many regions of the world, including countries across Africa, the Americas, Europe, and Asia [4, 5]. Orf disease has caused substantial economic losses to the livestock industry and has emerged as a global problem [6]. In China, small ruminant farming represents an important source of income for rural households, and outbreaks of Orf disease have been reported in Xinjiang, Tibet, Liaoning, and Gansu provinces [7–9]. Due to the self-limiting nature of the disease, its impact has often been underestimated. However, Orf disease is zoonotic and can be transmitted to humans, raising concerns for public health [10, 11].

The morbidity and mortality of Orf disease are quite variable and low in adult sheep and goats, while these are high in newborn kids and lambs [12, 13]. The ulceration and pustular dermatitis on the oral mucosa and lips prevent suckling or feeding in lambs and kids, leading to emaciation, secondary bacterial infections, or even death [14]. However, the reasons for the high morbidity of Orf disease in newborn lambs or kids remain unclear. The risk of viral transmission is higher during the grazing period, especially when animals feed on thorny plants or bushes, which create open wounds around the sensitive skin of the mouth, thereby providing entry for the virus [15]. Other transmission routes include direct contact with the infected animals, contaminated water tanks or feed troughs, fomites, and contaminated grass [16]. Interestingly, several reports have described the appearance of papules, pustules, and scabs around the lips and nostrils of suckling lambs and kids shortly after full-term parturition from Orf-disease-affected dams [17]. The newborn kids or lambs have very little chance of contact with the sharp objects that may cause open wounds, making conventional transmission routes insufficient to fully explain the early onset of infection. Therefore, existing transmission theories do not adequately account for the rapid development of clinical disease in neonates. Ma et al. [18] demonstrated the presence of active and infectious ORFV in the milk of asymptomatic goats, suggesting a possible source of infection for suckling kids. However, based on our observations, papular lesions may appear on the lips of kids as early as 3 days after birth, progressing to severe Orf disease within 1 week. Given the incubation period of ORFV, it is unlikely that such rapid disease progression results solely from postnatal exposure via milk or saliva. These observations underscore the need to investigate alternative transmission routes, including transplacental transmission. Therefore, we hypothesized that ORFV may be vertically transmitted from asymptomatic or carrier pregnant goats to their offspring during gestation. While previous studies have not conclusively demonstrated vertical transmission of ORFV, the present study provides compelling evidence supporting transplacental transmission from dams to their offspring during gestation.

The losses associated with the congenital transmission of the disease are potentially substantial; therefore, the development of effective preventive measures is crucial. Previous studies have shown that vaccination of pregnant dams can reduce the risk of Orf disease in their offspring. For example, Asin et al. reported a reduced prevalence of Orf disease in lambs after the vaccination of their pregnant dams [19]. After vaccination, maternal antibodies can be transferred across the placenta to the fetus, although the titer of the antibodies declines rapidly over time without active vaccination at the time of birth [20]. Therefore, maternal vaccination may represent a safe and effective strategy to decrease the burden of congenital infection.

In recent years, multiple outbreaks of Orf disease in lambs and kids have been reported [21, 22]. However, the mechanisms underlying the early onset and high morbidity of Orf disease in neonates, particularly the potential routes of transmission, remain poorly understood. This study aimed to investigate the vertical transmission of the virus from dams to their fetuses during gestation. In addition, this study evaluated potential strategies for the control and prevention of Orf disease in newborn kids.

## Materials and methods

The present study was carried out at the College of Veterinary Medicine, Northwest Agriculture and Forestry University, Yangling, China, after approval by the Research Ethics Committee of Northwest Agriculture and Forestry University, Yangling, China (IACUC2025-1102). The use of pregnant goats in this study was necessary to directly investigate the transplacental transmission of ORFV in goats and its control, which cannot be adequately modeled using in vitro or ex vivo systems. The number of animals included in this study was minimized to the lowest possible level required to achieve biological relevance, and sampling time points were selected on basis of gestational development and clinical observations to maximize data yield while reducing the use of animals.

### Collection of samples

Blood samples were collected from clinically healthy animals without clinical signs of Orf disease, including 95 female adult dairy goats and 66 kids residing at three different goat farms (A, B, and C), in Shaanxi province, China. The adult female goats and kids belonged to the Guanzhong dairy breed of goats and were aged between 1–4 years and 1–8 weeks, respectively. The maternal–fetal interface tissue samples were collected from another farm D. All the farms have intensive production systems. Immediately after kidding, the placental cotyledons (*n* = 24), umbilical cords (*n* = 22), amniotic fluid (*n* = 25), oral swab (*n* = 54), and blood of newborn kids (*n* = 22) were collected. The blood and oral swab samples were collected by following the guidelines of Cheng et al. [23] and Ma et al. [18]. Briefly, 2 mL of blood was drawn into 5 mL sterile syringes from the jugular vein and preserved at −20 °C in ethylenediaminetetraacetic acid (EDTA)-added vacutainers. The saliva was collected by placing and rotating the cotton swab on the tongue of newborn goats and placing cotton swabs in 2 mL sterile normal saline to refrigerate at 4 °C for 24 h. Finally, the supernatant of saliva was collected and preserved at −20 °C for later use. In addition, 5 mL amniotic fluid was drawn with a sterile syringe from the amniotic cavity. The placental cotyledons and umbilical cords were collected with sterile surgical scissors, rinsed three times with normal saline, and preserved in liquid nitrogen or fixed in 4% paraformaldehyde.

### Extraction of DNA and PCR amplification

The tissue samples were weighed before homogenization, and the genomic DNA was extracted from the tissues using standardized input masses and elution volumes by using the TIANamp Genomic DNA kit according to the manufacturer’s instructions. The concentration and purification of DNA were performed by a nanodrop method using a Thermo Scientific nanodrop machine. The conserved *B2L* gene was used to identify ORFV from genomic DNA by polymerase chain reaction (PCR) amplification. The forward and reverse primer sequences used to amplify the *B2L* gene were 5′-CGGAATTCAGTCCGCGAAGAAGTTTTTG-3′ and 5′-CCCTCGAGGCGAGTCCGAGAAGAATACG-3′, respectively [24]. The PCR reaction was run as previously described by Ma et al. [18]. Briefly, the 25 µL PCR reaction mixture contained 12.5 µL 2× Taq MasterMix (CWbiotech, Jiangsu, China), 2 µL template DNA, 9.5 µL double-distilled H_2_O (ddH_2_O), 0.5 µL forward primer, and 0.5 µL reverse primer. Genomic DNA was used as a positive control, while nuclease-free water was used as a negative control. The samples were run in a thermal cycler (Bio-Rad T100) for 5 min at 95 ℃ for initial denaturation, followed by 30 cycles of 30 s at 95 ℃ (denaturation), 30 s at 55 ℃ (annealing), and 60 s at 72 ℃ (extension). The final extension was performed for 10 min at 72 ℃, and then held at 4 ℃. All the PCR products having an amplicon size of 514 bp were separated by gel-electrophoresis in 2.0% agarose gels stained with SuperRed.

### Isolation of primary cells from the goat fetus

The primary cells of the goats were isolated by following the instructions of Ma et al. [15]. Ten healthy female adult goats (1 year old) were reared with a buck in the same pen, and two sodium cloprostenol injections (0.2 mg/injection) were administered intramuscularly into the buttocks of the goats. After 60 days, the pregnant goats were identified by using an ultrasonography machine. The uterus of these pregnant goats was dissected to collect the fetus and placenta under general anesthesia in a sterile environment. The fetus was removed from the placenta and rinsed three times with sterile phosphate buffer saline (PBS). The lip tissue of the fetus was used to prepare goat primary fetal lip cells, while the other body parts, after removing the head, limbs, and tails, were also used to prepare primary fetal cells. The small pieces of tissue (1 mm^3^) were digested with collagenase IV (Gibco, Thermo Fisher Scientific, Waltham, MA, USA) while incubating at 37 °C and 5% CO_2_ for 12 h. After digestion, the cells were filtered through a 200-mesh nylon filter, washed twice with serum-free Dulbecco's modified Eagle’s medium (DMEM)/F12 medium, and resuspended in 10% DMEM/F12 medium supplemented with 10% fetal bovine serum and 1% penicillin–streptomycin (10000 units) for later use.

### Isolation of ORFV from placental cotyledons and in vitro infection of fetal primary cells

The ORFV-positive placental cotyledon was ground with 5 mL PBS and 1 mL penicillin–streptomycin (10000 units; Hyclone) using a tissue homogenizer. The mixture was centrifuged at 6000 × *g* for 3 min, and the supernatant was collected and stored at −80 ℃. Further, the collected supernatant was freeze-thawed three times. Finally, the supernatant was filtered through 0.45 and 0.22 μm filter papers. The fetal lips and primary cells were incubated with the filtrate containing ORFV for 1 h at 37 ℃ in 5% CO_2_. Then, 10 mL minimum essential medium supplemented with 2% fetal bovine serum was added to the cells, and the cytopathic effect (CPE) was observed after five generations of ORFV or 72 h postinfection of the culture under an inverted microscope (Axio Observer, Zeiss, Germany).

### Immunofluorescence

The preserved placental cotyledons in 4% paraformaldehyde were rinsed and dehydrated with 15% and 30% sucrose solutions. The freezing microtome (POLAR DM, Japan) was used to cut 5 μm-thick tissue slices. The infected primary cells of goats were also fixed with 4% paraformaldehyde for 30 min at room temperature. The frozen tissue slices or fixed cells were permeabilized with 0.5% Triton X-100 for 15 min at room temperature, blocked with donkey serum (Abcam, Cambridge, UK) for 2 h, and stained with ORFV monoclonal antibody (Santa Cruz Biotechnology, Dallas, Texas, USA) overnight at 4 ℃. These cells and slices were incubated with donkey anti-mouse antibody IgG heavy and light chains (H&L) (Alexa Fluor® 488; Abcam) for 2 h at room temperature, stained with 4′,6-diamidino-2-phenylindole (DAPI) solution (Roche, Basel, Switzerland), and placed in a dark place for 30 min. Laser scanning confocal microscopy (LEICA TCS SP8) or an inverted microscope (Axio Observer, Zeiss, Germany) was used to observe these cells.

### Establishment of ORFV *B2L* standard curve for real-time PCR

The DNA of ORFV was extracted from the primary goat lip cells infected with ORFV and amplified by PCR according to the same method as described in the “Extraction of DNA and PCR amplification” section. The amplified DNA products were purified by using a TIAN Midi purification kit (Tiangen Biotech, Beijing, China) according to the manufacturer’s instructions. The purified DNA was constructed into the vector using the pGM-T cloning kit (Tiangen Biotech, Beijing, China). The selected positive plasmids were sequenced (Sangon Biotech, Shanghai, China) and compared with the online available whole genome sequence of ORFV *B2L* by using the National Center for Biotechnology Information (NCBI) Genbank database Basic Local Alignment Search Tool (BLAST). The standard DNA was extracted from the DNA-identified positive plasmid by the TIANprep mini plasmid kit (Tiangen Biotech, Beijing, China), and the standard DNA concentration was determined using a NanoDrop 2000 system (Thermo Fisher Scientific, USA). As previously reported by Wang et al. [24], the concentration of DNA was calculated into the number of copies (copies/µL), and ten-fold serial dilutions with double-distilled water were performed to prepare 1 × 10^1^ copies/μL from 1 × 10^8^ copies/μL.

For the quantification of viral load in samples, the real-time PCR was performed using SuperReal Premix Plus (SYBR green). The viral load was normalized and expressed as genome copies per mg of tissue, while fluid samples were reported as copies/mL to ensure comparability across different sample types. The conserved *B2L* gene of ORFV primer sequence was used for real-time PCR (forward primer 5′-GGGCTCTACTCCACCAACAA-3′ and reverse primer 5′-CGAGTCCGAGAAGAATACGC-3′) [24]. Briefly, the 20 μL real-time PCR reaction mixture contained 10 μL of 2× SuperReal Premix Plus, 0.6 μL of each 10 μM primer, 2 μL of template DNA, and 6.8 μL of ddH_2_O. Real-time PCR was performed using a real-time PCR detection system (Bio-Rad, Hercules, CA, USA) with a recommended two-step PCR reaction procedure, such as 15 min of denaturation at 95 ℃ followed by 40 cycles of denaturing at 95 ℃ for 10 s, annealing, and extension at 60 ℃ for 32 s. The obtained cycle threshold (Ct) values measured by DNA of different concentrations were used as the vertical coordinate, and log (copy number of template DNA) was used as the horizontal coordinate to draw standard curves. The Ct values of different samples were substituted into the above standard curve formula to calculate the copy number.

### Preparation of inactivated vaccine and vaccination of pregnant goats

The ORFV FX strain isolated from scabs of infected goats in Fengxiang, Shaanxi province, China, was already preserved in our laboratory and used for the preparation of an inactivated vaccine. The purified virus (10^7.8^ 50% tissue culture infectious dose (TCID_50_)/100 μL) was inactivated by β-propiolactone (Solarbio, Beijing, China) with a final concentration of 0.1% for 24 h. The virus was incubated at 37 ℃ for 2 h to hydrolyze β-propiolactone. The aluminum salt as an adjuvant was mixed with the inactivated virus at a ratio of 1:1, and the prepared vaccine was stored at 4 ℃ for later use. To evaluate the effect of the inactivated vaccine, a goat farm (farm E) with pregnant goats was selected and randomly divided into two groups: a vaccinated group and a non-vaccinated group. The first group received two subcutaneous doses of the inactivated ORFV vaccine (2 mL per dose), with the second dose administered after a 1-month interval during the early-stage and mid-stage of gestation, while the second group (control group) received two doses of normal saline subcutaneously instead of the vaccine with the same vaccination interval. During the time of kidding, the blood from the dam, umbilical cord, and newborn kids were collected for molecular confirmation of the virus.

### Statistical analysis

All the data were recorded in Microsoft Excel 365. A chi-squared (*χ*^2^) test was used to determine the association between ORFV positivity and the different sample types. Relative risk (RR) values with 95% confidence intervals (CIs) were calculated to quantify the likelihood of ORFV detection across groups. The Fisher’s exact test was also applied where appropriate to calculate the odds ratios. All analyses were performed using standard statistical methods in GraphPad Prism 10.6.1, and *p* < 0.05 was considered statistically significant.

## Results

### Detection of ORFV between maternal goats and kids and their association

**Figure 1:**
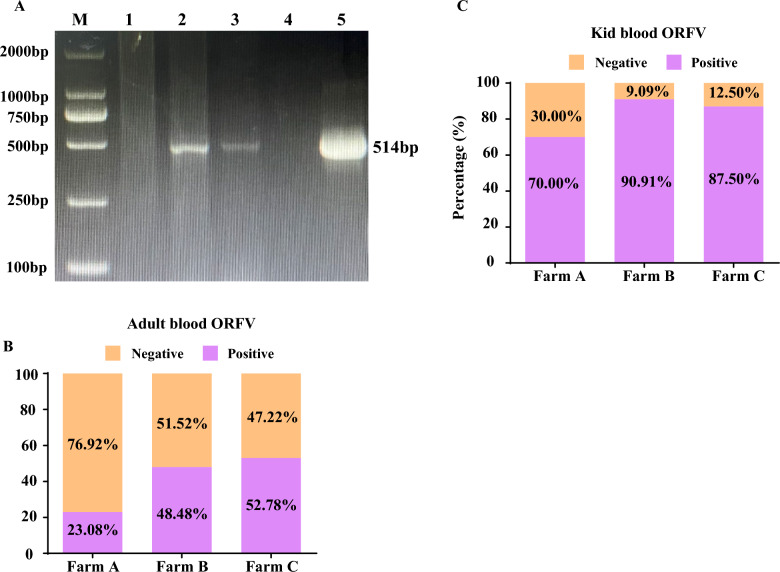
**Detection of ORFV in dams and their kids**. **A** Representative PCR analysis of the *B2L* gene in blood samples from adult goats and kids. M: DNA marker; lane 1: blood of an ORFV-negative kid; lane 2: blood of an ORFV-positive kid; lane 3: blood of an ORFV-positive maternal goat; lane 4: double-distilled water as negative control (no DNA template); lane 5: DNA from ORFV-infected cells as positive control. **B**, **C** ORFV detection rates in the blood of maternal goats (**B**) and kids (**C**) from three goat farms.

The blood samples of 161 animals were tested for the detection of ORFV, and the molecular identification of ORFV is presented in Figure 1A, Additional file 1, and Table 1. The overall positive detection rate of ORFV in goats and their kids at three farms was 59.63% (96/161; 95% CI: 51.61–67.27%). The highest infection rate was detected at farm C at 66.67% (40/60; 95% CI: 53.31–78.31%), followed by farm B at 65.45% (36/55; 95% CI: 51.41–77.76%) and farm A at 43.48% (20/46; 95% CI: 28.93–58.89%). The overall occurrence of ORFV at three farms in dams was 43.16% (41/95; 95% CI: 33.03–53.72%), and in kids was 83.33% (55/66; 95% CI: 72.13–91.37%). In dams, the highest infection rate was observed at farm C at 52.78% (19/36; 95% CI: 35.48–69.59%), followed by farm B at 48.48% (16/33; 95% CI: 30.79–66.45%) and farm A at 23.08% (6/26; 95% CI: 8.97–43.64%). Similarly, the highest infection rate in kids was observed at farm B at 90.91% (20/22; 95% CI: 70.83–98.87%), followed by farm C at 87.50% (21/24; 95% CI: 67.63–97.34%) and farm A at 70.00% (14/20; 95% CI: 45.72–88.10%). The distribution of ORFV-positive and -negative adult goats and their kids at three farms is shown in Figures 1B, C. The relative risk values across farms ranged from 0.33 to 0.60, demonstrating that adult goats were substantially less likely to be infected than kids. In contrast, the reciprocal of RR ranged from 1.66 to 3.03, suggesting that kids had approximately two to three times higher risk of ORFV infection compared with adults. Overall, kids exhibited approximately a 92% higher likelihood of infection compared with adults. These results clearly indicate that younger animals are more susceptible to ORFV infection.

**Table 1:** **Detection of ORFV in three goat farms**

Location	Animals	Samples examined	Samples positive (%)	Statistical analysis					
				*χ*^2^	*p*-value	RR			
						RR	95% CI	Reciprocal of RR	95% CI
Farm A	Dams	26	6 (23.08)	10.13	0.0015	0.33	0.15–0.67	3.03	1.50–6.61
	Kids	20	14 (70.00)						
Farm B	Dams	33	16 (48.48)	10.51	0.0012	0.53	0.35–0.76	1.89	1.31–2.84
	Kids	22	20 (90.91)						
Farm C	Dams	36	19 (52.78)	7.81	0.0052	0.60	0.41–0.85	1.67	1.18–2.41
	Kids	24	21 (87.50)						
Overall	Dams	95	41 (43.16)	26.11	0.0001	0.52	0.40–0.67	1.92	1.51–2.52
	Kids	66	55 (83.33)						

CI: confidence interval, RR: relative risk.

### Transplacental transmission of ORFV

**Figure 2:**
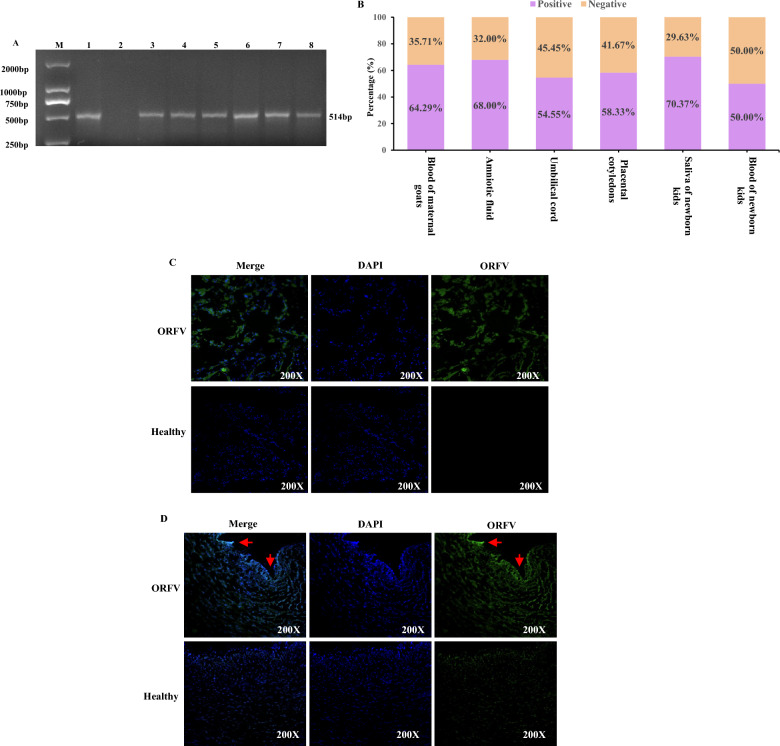
**Presence of ORFV in maternal–fetal interface tissues**. **A** PCR analysis of representative maternal and neonatal samples. Lane 1: DNA from ORFV-infected cells as a positive control; lane 2: double-distilled water as negative control (no DNA template); lane 3–8: representative ORFV-positive samples, including the blood of a maternal goat (lane 3), amniotic fluid (lane 4), umbilical cord (lane 5), placenta (lane 6), saliva of a newborn kid (lane 7), and blood of a newborn kid (lane 8). **B** Detection rates of ORFV in the blood of dams and kids, and in maternal–fetal interface tissues. **C**, **D** Immunofluorescence staining of placenta (**C**) and umbilical cord (**D**) tissues from ORFV-infected and healthy goats. ORFV is shown in green, and cell nuclei are stained with DAPI (blue).

The screening of the blood of pregnant goats was performed to determine the transmission of the virus to their offspring. Immediately after parturition, the samples (placental cotyledons, umbilical cords, and amniotic fluids) can be detected positive for ORFV (Figure 2A; Additional file 2). Similarly, the blood and saliva of newborns were collected before their first suckling and could be found to be positive for the virus. The blood-positive rate in pregnant goats was found to be 64.29% (27/42; 95% CI: 48.02–78.44%), and that for neonatal kids was 50.00% (11/22; 95% CI: 28.22–71.77%). The positive rates of the samples are presented in Table 2 and Figure 2B. To further verify the presence of ORFV in the maternal–fetal interface tissues, the immunofluorescence staining of the samples was performed. The result revealed the presence of ORFV in these tissues, and the endothelial lining of the blood vessels was found to be strongly positive for the virus. It indicated that both placental cotyledons (Figure 2C) and umbilical cords (Figure 2D) were infected with the pathogen, with the infection being more evident to the blood vessel lining of the umbilical cord.

**Table 2:** **Raw numbers of positive samples for ORFV and ORs for positivity**

Type of samples	Samples examined	Number of positive samples	Positive rate (%)	OR (95% CI)
Blood of maternal goats	42	27	64.29	1 (reference)
Amniotic fluid	25	17	68.00	1.18 (0.42–3.63)
Umbilical cord	22	12	54.55	0.66 (0.24–1.87)
Placental cotyledon	24	14	58.33	0.77 (0.29–2.11)
Saliva of neonatal kids	54	38	70.37	1.31 (0.54–3.18)
Blood of neonatal kids	22	11	50.00	0.55 (0.19–1.51)

CI: confidence interval, ORs: odds ratios.

**Figure 3:**
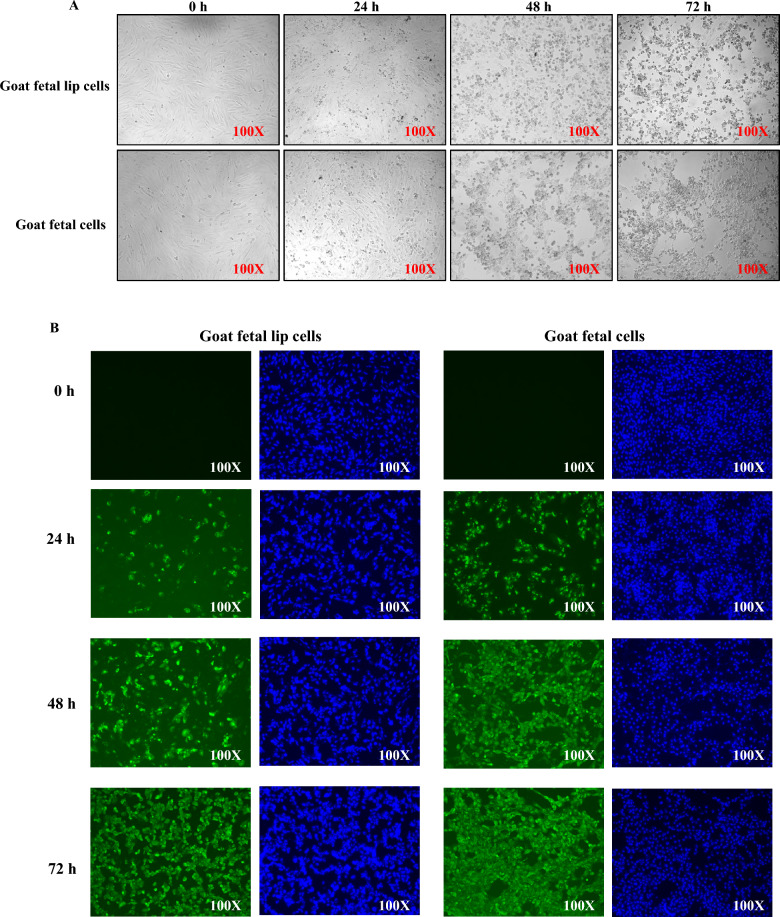
**Pathogenicity of ORFV isolated from the placental tissues**. Primary goat fetal lip cells and goat fetal cells were infected with ORFV. Cytopathic effects (**A**) and immunofluorescence detection of ORFV (**B**) were observed at different time points postinfection. For **B**, ORFV is shown in green, and cell nuclei are stained with DAPI (blue).

To further verify the transmission of ORFV from the dam to its offspring through the placenta, the goat’s primary fetal and lip cells were infected with ORFV isolated from the ORFV-positive placental cotyledons and incubated to establish the infection. Some cells were found to be round after 24 h of infection. With further advancement in the infection time (48 h), the shape of more cells changed, and they became round and clustered together. Similarly, after 72 h of infection, a large number of cells were torn (Figure 3A). Furthermore, cellular immunofluorescence was performed to determine the proliferation profile in these cells or the detection of ORFV-infected cells. The results indicated that the ORFV-specific green fluorescence gradually increased as the infection time was prolonged. The green fluorescence was detected more in the goat primary fetal cells as compared with the lip cells (Figure 3B). These results demonstrated that ORFV can infect all the primary fetal or lip cells of the goats after 72 h of the establishment of the infection, while the virus proliferates more rapidly in the goat’s primary fetal cells. These findings demonstrated the presence of ORFV in placental tissues and supported its potential transfer from dams to fetuses through the placenta.

### Determination of viral load

**Figure 4:**
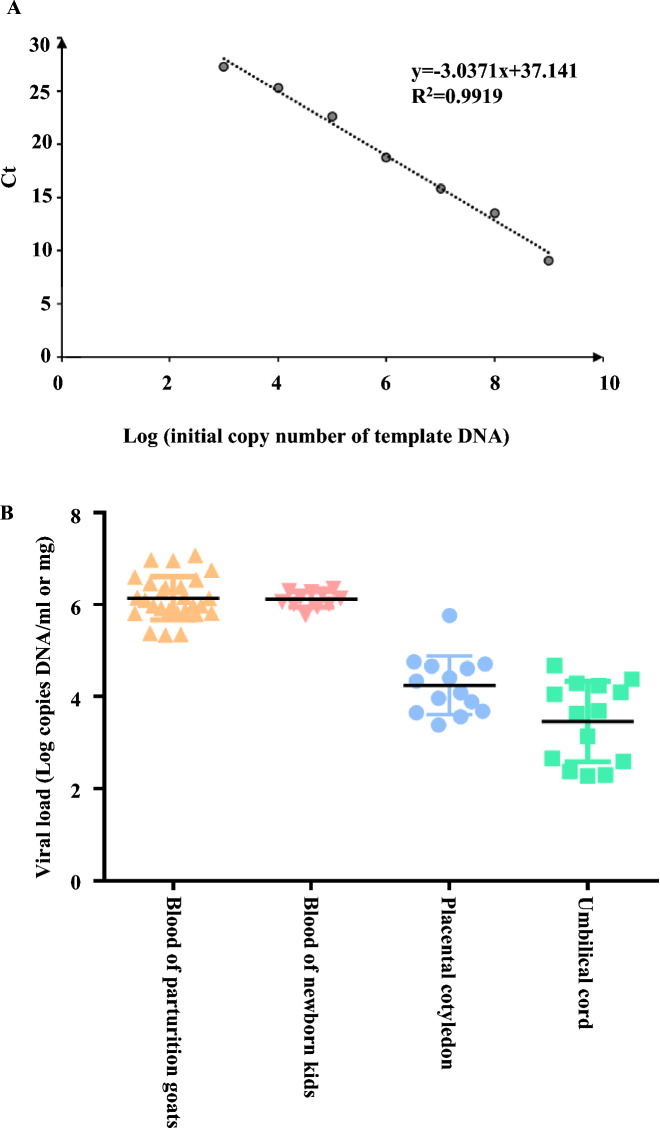
**Quantification of ORFV viral load in different samples**. **A** Standard curve for absolute quantification of ORFV by real-time PCR (formula: *y* = −3.0371*x* + 37.141). **B** Viral loads of ORFV in different tissues from dams and kids.

To estimate the range of ORFV load in the maternal–fetal interface tissues that can be transmitted from pregnant goats to neonatal kids through the placenta, the level of viral loads in the blood of dams, neonatal kids before suckling, and other tissues was determined. Firstly, the absolute quantitative standard curve of ORFV was developed by real-time quantitative PCR. The standard DNA was obtained from the ORFV FX strains stored in our laboratory. Ct values of 1 × 10^1^ copies/μL to 1 × 10^8^ copies/μL of standard DNA were used to draw the standard curve, and the formula of the standard curve was calculated as: *y* = −3.0371*x* + 37.141 (Figure 4A). Absolute quantitative results showed that the ORFV load in 27 blood samples of pregnant goats ranged from 10^5.34^ copies/mL to 10^7.07^ copies/mL, with an average of 10^6.14^ copies/mL, while in that of kids’ blood samples (*n* = 11), it ranged from 10^5.77^ copies/mL to 10^6.35^ copies/mL, with an average of 10^6.11^ copies/mL. The virus load in other samples, such as placental cotyledons and umbilical cord, ranged from 10^3.38^ copies/mg to 10^5.76^ copies/mg, with an average of 10^4.25^ copies/mg, and from 10^2.28^ copies/mg to 10^4.68^ copies/mg, with an average of 10^3.50^ copies/mg, respectively (Figure 4B).

### The inactivated vaccine protects newborn goats by preventing the transmission of the virus to the fetus through the placenta

The effect of the inactivated ORFV vaccine was evaluated in parturient goats, umbilical cord blood, and kids. The vaccination of pregnant goats significantly reduced the vertical transmission rate of ORFV, with the positive rate decreasing from 90.00% in non-vaccinated dams to 56.14% in vaccinated dams, in umbilical cord blood from 71.43% in non-vaccinated dams to 14.29% in vaccinated dams, and in kids from 36.67% in non-vaccinated dams to 0% in vaccinated dams (Table 3). The results of relative risk analysis for the vertical transmission of virus after vaccination showed that the RR of parturition goats was 0.63 (95% CI: 0.52–0.74), the RR of umbilical cord blood was 0.20 (CI: 0.12–0.32), and the RR of kids was 0.00 (CI: 0.00–0.50). Vaccination of pregnant goats reduced the likelihood of infection by 37.6% in the does and by 80% in umbilical cord blood. Likewise, the vaccine conferred complete or nearly complete protection to neonatal kids against ORFV. The results suggested that vaccination significantly decreased the possibility of vertical transmission of ORFV from dams to kids in goats.

**Table 3:** **Effect of vaccination on ORFV detection in dams and kids**

Source	Non-vaccinated		Vaccinated		Statistical analysis					
					*χ*^2^	*p*-Value	RR			
							RR	95% CI	Reciprocal of RR	95% CI
	Samples examined	Samples positive (%)	Samples examined	Samples positive (%)						
Parturient goat blood	80	72 (90.00)	114	64 (56.14)	25.72	0.0001	1.60	1.35–1.94	0.63	0.52–0.74
Umbilical cord blood	70	50 (71.43)	112	16 (14.29)	60.86	0.0001	5.00	3.16–8.11	0.20	0.12–0.32
Kid blood	60	22 (36.67)	114	0 (0.00)	47.85	0.0001	∞	2.0–∞	0.00	0.00–0.50

CI: confidence interval, RR: relative risk.

## Discussion

This study provides compelling evidence supporting the vertical transmission of ORFV from infected dams to their offspring through the placenta in goats. This study also demonstrates that vaccination during early or mid-gestation can effectively reduce vertical transmission. These findings have important implications for the epidemiology, prevention, and control of ORFV in small ruminants.

Orf disease is endemic in China, with reported outbreaks in multiple provinces. For example, Zhang et al. [25] described an outbreak in a goat herd in Jiangxi Province in 2021, and Wang et al. [26] reported an outbreak in Anhui Province in 2018. Similarly, Li et al. [27] reported disease occurrence in sheep herds in Northeast China. Considering the frequent outbreaks of Orf disease worldwide, including China, it is important to clearly understand the routes of transmission to neonatal kids or lambs, which are more severely affected than adults, and to explore potential preventive strategies [13]. Moreover, Orf disease is zoonotic and may infect workers closely associated with small ruminants. In China, ORFV transmission from goats to two humans with clinical symptoms has been reported in Gansu Province [28]. Unlike previous studies that focused primarily on the outbreaks [25–27], this study emphasizes a novel transmission route and the effectiveness of an inactivated vaccine in preventing the vertical transmission of ORFV in goats. The detection of viral DNA in the blood and saliva of newborns, as well as in maternal–fetal interface tissues, demonstrates that ORFV can cross the placental barrier. This evidence supports the hypothesis that in utero infection may contribute to the early onset of ORFV infection in newborn kids, which cannot be fully explained by postnatal exposure through milk or saliva [18]. The detected viral loads, ranging from 10^2.28^ to 10^7.07^ copies/mg (tissue samples) or copies/mL (fluid samples), are sufficient to cause disease.

Traditionally, ORFV is transmitted primarily through direct contact, fomites, or environmental contamination [15]. However, our results indicate that infected dams can act as reservoirs of infection and transmit the virus directly to their offspring. Fortunately, no abortion, stillbirth, or congenital deformity was observed in ORFV-infected neonatal kids. Papules around the lips and nostrils were observed in 3–7-day-old suckling kids born to ORFV-infected dams, and negative impacts such as reduced weight gain and economic losses in severe cases should not be overlooked [14, 17]. These findings highlight the importance of monitoring and vaccinating pregnant does in large-scale sheep and goat farms.

To control the outbreak of Orf disease in sheep and goats, it is necessary to develop a safe, effective, and long-acting vaccine. The commercially available live attenuated vaccines provide partial protection [29]. Studies have shown that ORFV can act as an adjuvant to enhance immune responses [30], which supports our findings that inactivated vaccination of dams may stimulate protective maternal immunity. Vaccination reduced ORFV detection in neonatal kids, effectively eliminating detectable viremia. Moreover, no clinical signs or Orf-disease-like lesions were observed following vaccination, highlighting its effectiveness. The most likely mechanism is that vaccination might have stimulated an effective maternal immune response, which either prevented viremia in dams or enabled the transfer of protective maternal antibodies to the offspring, thereby preventing the vertical viral transmission. However, in this study, serological analyses, e.g., enzyme-linked immunosorbent assay (ELISA) or virus neutralization assay to confirm antibody titers in dams or kids, were not performed, and thus, this mechanism remains hypothetical.

Understanding vertical transmission of pathogens has important implications for disease management, highlighting the need to monitor infection status in pregnant animals to prevent in utero spread [31]. Vaccination before or during gestation may reduce the risk of transmission to offspring. Additional measures, such as isolating high-risk neonates, providing an uncontaminated environment, and targeted surveillance of both dams and newborn animals, may further limit the spread of infection [32, 33]. Future studies should quantify antibody titers and evaluate the efficacy of the inactivated ORFV vaccine, assess immune responses in placebo groups, and investigate the pathological and immunological consequences of vertical ORFV infection in neonates, including effects on viability, growth, and susceptibility to secondary infections.

## Conclusion

ORFV can infect the fetus during gestation and be transmitted from the dam through the placenta. Vaccination of pregnant goats can effectively reduce vertical transmission and prevent neonatal infection. This study provides important evidence of vertical ORFV transmission in goats and demonstrates an effective strategy for the prevention and control of ORFV infection in newborn kids.

## Supplementary Information

Additional file 1.

Additional file 2.

## Data Availability

The data presented in this study are available within the article.
