# Supplementary material for: Vertical transmission of Orf virus in goats and its prevention
Source: Vet Res. 2026 Feb 25;57:47. doi: 10.1186/s13567-026-01714-0 (PMC13041380; doi:10.1186/s13567-026-01714-0)
Supplement: Supplementary file 1 — Additional file 1. [file 13567_2026_1714_MOESM1_ESM.docx]

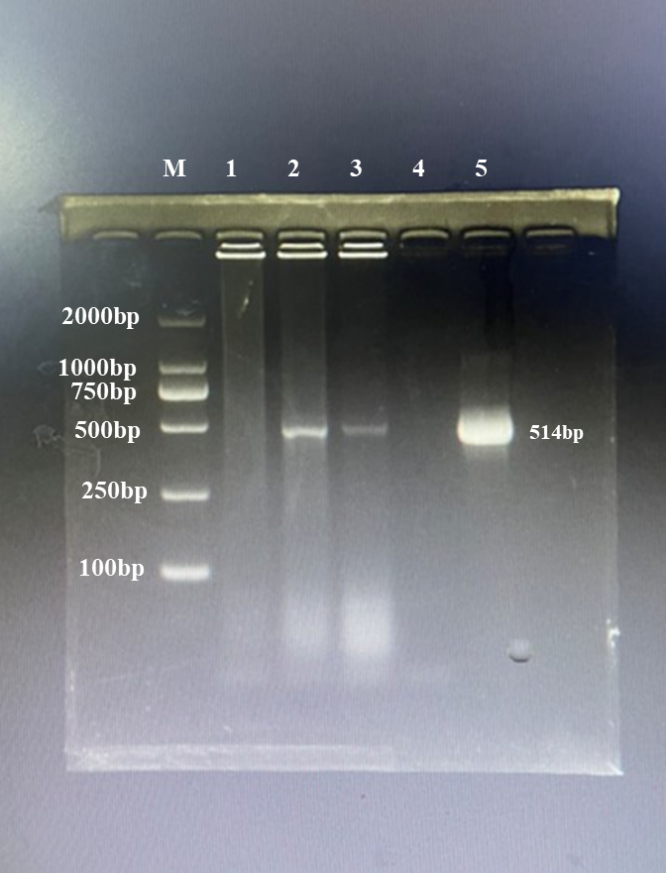


**Additional file 1: Uncropped agarose gel image of Figure 1A.** M: DNA marker; lane 1: blood of an ORFV-negative kid; lane 2: blood of an ORFV-positive kid; lane 3: blood of an ORFV-positive maternal goat; lane 4: double-distilled water as negative control (no DNA template); lane 5: DNA from ORFV-infected cells as positive control.
