# Supplementary material for: Vertical transmission of Orf virus in goats and its prevention
Source: Vet Res. 2026 Feb 25;57:47. doi: 10.1186/s13567-026-01714-0 (PMC13041380; doi:10.1186/s13567-026-01714-0)
Supplement: Supplementary file 2 — Additional file 2. [file 13567_2026_1714_MOESM2_ESM.docx]

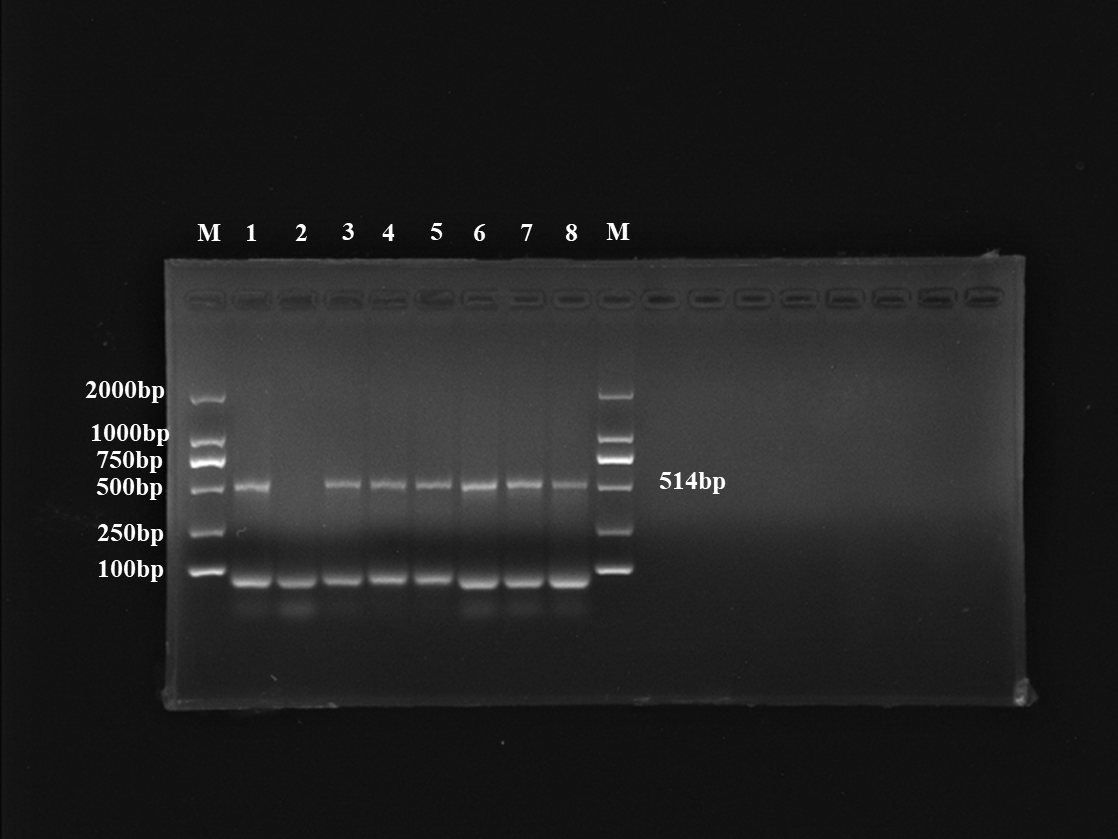


**Additional file 2: Uncropped agarose gel image of Figure 2A.** Lane 1: DNA from ORFV-infected cells as a positive control; lane 2: double-distilled water as negative control (no DNA template); lane 3-8: representative ORFV-positive samples, including the blood of a maternal goat (lane 3), amniotic fluid (lane 4), umbilical cord (lane 5), placenta (lane 6), saliva of a newborn kid (lane 7), and blood of a newborn kid (lane 8).
